# Integrating Global Surveillance, Local Action, and Innovative Stewardship Against Antimicrobial Resistance

**DOI:** 10.3390/antibiotics14080835

**Published:** 2025-08-18

**Authors:** Jesús Ruiz-Ramos

**Affiliations:** 1 Pharmacy Department, Hospital de la Santa Creu i Sant Pau, Sant Antoni Maria Claret 167, 08025 Barcelona, Spain; jrzrms@gmail.com; 2 Institut de Recerca Sant Pau (IR SANT PAU), Sat Quintí 77-79, 08041 Barcelona, Spain

The silent pandemic of antimicrobial resistance (AMR) represents one of the gravest threats to global health of the 21st century [1]. The time when bacterial infections were easily cured is coming to an end, and with it, modern medicine faces the risk of even the most common procedures becoming lethal. In this global struggle, two core strategies have emerged as our principal pillars of defense: vigilant surveillance to understand the threat and proactive antimicrobial stewardship to preserve our therapeutic arsenal. This Special Issue of *Antibiotics*, “Optimizing Antimicrobial Use: Antimicrobial Stewardship and Surveillance”, brings together a collection of articles that explore the depth and breadth of these pillars. From the global scale of drug development to the granular details of hospital logistics, and from the chaotic frontline of the emergency department to the often-overlooked environmental reservoirs of resistance, these contributions paint a comprehensive picture of our current challenges and future research directions.

No effective antimicrobial strategy can be built without a foundation of vigilant surveillance. It provides the essential data for monitoring resistance trends, guiding clinical therapy choices, and informing the development of next-generation agents. Two articles in this Special Issue exemplify the power of large-scale, international surveillance programs like ATLAS. The work by Piérard et al. [2] provides a critical evaluation of aztreonam–avibactam, a combination agent with the potential to address one of our most urgent threats: metallo-β-lactamase (MBL)-producing *Enterobacterales*. Their comprehensive analysis, spanning 2016 to 2020, demonstrates the compound’s potent in vitro activity against these highly resistant pathogens across different wards and infection sources. This type of global surveillance is indispensable; it not only validates the clinical development pipeline but also provides the data necessary to strategically deploy new weapons against our most formidable microbial foes. Similarly, the study by Kuraieva et al. [3] uses the ATLAS platform to reaffirm the role of an established agent, ceftaroline, against key pathogens in skin and soft tissue infections (SSTIs). By demonstrating its sustained potent activity against both methicillin-susceptible and -resistant *Staphylococcus aureus* (MSSA and MRSA) and β-hemolytic streptococci, their work underscores how continuous monitoring helps maintain confidence in and refine the use of our existing formulary for specific clinical syndromes.

While global surveillance provides the map, local surveillance provides the ground truth. The study by Venegas-Esquivel et al. [4] masterfully illustrates this by conducting a point-prevalence survey of antibiotic use in six second-level hospitals in Mexico. Their findings are a stark reminder of the realities on the ground: an overall antibiotic use prevalence of 61%, with low adherence to guidelines (53.9%) and a predominance of WHO “Watch” group antibiotics, particularly for indications like surgical prophylaxis where “Access” group agents are preferred. This work highlights the critical need for local data to identify specific targets for stewardship interventions. It is this granular, institution-level surveillance that transforms broad principles into focused, impactful action, revealing the precise gaps where stewardship efforts are most needed.

Surveillance and antimicrobial stewardship programs (ASPs) are complementary functions: one identifies resistance trends, and the other dictates the clinical response. This series of articles examines the practical application of stewardship, from its implementation at the bedside to the development of innovative frameworks. Within this scope, the emergency department (ED) represents a uniquely challenging environment for successful ASP implementation. The review by Ruiz-Ramos et al. [5] provides a thoughtful exploration of this environment, characterized by diagnostic uncertainty, time pressure, and high patient turnover. They delineate the specific challenges that hinder traditional stewardship models while highlighting proven interventions—such as rapid diagnostic tests, culture follow-up programs for discharged patients, and prospective audit with feedback—that can succeed. This research underscores the need for context-specific stewardship, as any effective strategy must be adapted to a clinic’s unique operational pressures.

The scope of this challenge, however, extends far beyond hospital walls. Adopting a “One Health” paradigm is essential, as it provides a framework for tackling AMR as a shared problem across human, animal, and environmental ecosystems. The study by Kim et al. [6] offers a compelling case study from this perspective, investigating AMR in *E. coli* isolated from diverse environmental sources in Central Virginia, including animal feces, drainage water, and wastewater treatment plants (WWTPs). Their discovery that WWTPs harbored the highest prevalence of AMR, with one isolate resistant to seven antimicrobials, identifies these facilities as crucial “hotspots” for resistance. This research powerfully demonstrates that a comprehensive stewardship and surveillance strategy must look upstream to the environmental sources that seed our communities and hospitals with resistant organisms.

Looking ahead, the evolution of stewardship theory must be matched by an evolution in practice. This means developing more sophisticated ways to implement these strategies and measure their real-world impact. The innovative work by Leistner et al. [7] introduces the concept of “Logistic Stewardship”, challenging a fundamental metric of many ASPs: antibiotic consumption. By meticulously comparing logistical data (Defined Daily Doses, or DDDs) with actual patient prescriptions (Prescribed Daily Doses, or PDDs), they uncovered a significant discrepancy, with logistical data overestimating true consumption by over 30%. They identified hidden logistical patterns like inter-ward “cross-supply” and “hoarding” that distort our picture of antibiotic use. This is a crucial insight; if our core metric is flawed, our interventions may be misdirected. Their findings suggest that by focusing on optimizing specific areas like surgical prophylaxis and ensuring timely therapy re-evaluation, ASPs can achieve a more significant and accurately measured impact. This calls for a paradigm shift, integrating logistical expertise into clinical ASP teams to ensure our efforts are both efficient and effective.

In conclusion, the collection of articles in this Special Issue powerfully demonstrates that optimizing antimicrobial use is a multifaceted endeavor. It requires a seamless integration of surveillance across all scales—global, local, and environmental—to generate the intelligence we need. This intelligence must then fuel dynamic and innovative stewardship programs that are not only evidence-based but also context-aware and logistically sound. Ultimately, protecting the long-term efficacy of antimicrobials will depend on sustained collaboration and a commitment to developing new approaches. This collection offers a realistic appraisal of the current challenges while also outlining a viable framework of integrated strategies for moving forward successfully.

## Abbreviations

The following abbreviations are used in this manuscript:

AMR	Antimicrobial Resistance
WWTPs	Wastewater Treatment Plants
ASP	Antimicrobial Stewardship Program
DDD	Defined Daily Dose
